# *The Organon* and Materia Medica Club of the Bay Cities of California

**Published:** 1897-04

**Authors:** W. E. Ledyard

**Affiliations:** B. A., M. B., M. R. C. S. Eng., Secretary


W. E. LEDYARD, B. A., M. B., M. R. C. S. Eng.,
*Secretary.*

---

### *THE ORGANON* AND MATERIA MEDICA CLUB OF THE BAY CITIES OF CALIFORNIA.

The regular semi-monthly meeting was held at the office of Dr. George H. Martin, 606 Sutter Street, San Francisco, on Friday evening, December 4th, 1896.

The meeting was called to order by the President, Dr. J. M. Selfridge, at 8 P. M.

Members present: Drs. J. M. and C. M. Selfridge, George H. Martin, G. J. Augur, and W. E. Ledyard.

The motion that that portion of the By-Laws which requires the Secretary to notify the members at least one week in advance of an election be stricken out was adopted.

In behalf of the Board of Censors Dr. Martin reported favorably on the name of Dr. S. G. Tucker, 1317 Clay Street, Oakland, as a Senior Member of the Club; also on that of Dr. C. J. Holmgren, 1618 Folsom Street, San Francisco.

Both of the above, after motions to that effect, the President then declared unanimously elected as Senior Members of the Club.

The Treasurer (producing his cash book) showed only fifteen cents on hand.

On motion an assessment of fifty cents was levied on each member.

Sections 231 to 240 of *The Organon* were then read and discussed.

Dr. Martin, referring to the last clause of Section 231, said: There are in mental conditions, at times, cases in which the mental depression will be prominent and the physical symptoms not pronounced. Then the reverse of this may hold good, or the physical and mental become very much mixed up.

This alternation of the mental and bodily symptoms is a wonderful fact of Nature. This is especially so in hysterical cases.

In these cases we must take into consideration the pathological condition.

Dr. J. M. Selfridge—I do not agree with you as to the necessity of considering the pathological condition. *The totality of the symptoms must always be our guide in the selection of the medicine.* However, I admit that the cases referred to are very puzzling.

Dr. Martin—It is absolutely necessary to take in the whole range of symptoms.

Dr. Ledyard—In cases in which symptoms alternate we would expect the greatest relief from medicines which produce alternating symptoms, such as *Platina, Ignatia, Pulsatilla*, Asafetida, etc.

Dr. J. M. Selfridge—It would seem (from this Section 232) that Hahnemann was in the habit of alternating.

Dr. Martin—The meaning is rather that Hahnemann gave the antipsoric medicine as long as it was indicated, and *then* gave the antisyphilitic.

Dr. Augur reported a case of emphysema, in which a nervous derangement, caused by the receipt of bad news, called for *Ignatia,* before the antipsoric treatment could be resumed. Before studying Homeopathy he had heard of homeopathic physicians curing intermittent fevers, and didn't believe it. However, since beginning to practice

Homeopathy he has cured quite a number of such cases with the 200th potency.

Dr. Ledyard reported a case of asthma following the suppression of chills by Quinine. The asthma had affected the patient for forty-five years. After taking Quinine frequently in massive doses of the crude drug, the chills stopped; but, *from that time on the patient suffered from asthma.* He made known the nature of the case to the patient, and also the advisability of bringing back the chills, stating that when the chills had been brought back, the asthma would either disappear or would be amenable to treatment. Having the patient's concurrence, he commenced the treatment by giving *one dose of Chin-sulph.* (*Quinine*) *200th,* from time to time, repeating the dose. After treating the case for some time a slight afternoon *fever,* with dryness of the lips and thirst, put in an appearance. The case was persevered in, *an occasional dose of very highly potentized Sulphur* being exhibited until *the yawning and stretching with the identical old-time chills, and even the bitter taste of the Quinine returned in full force.*

After this attack the asthma was reduced to a minimum, and from that day to this has troubled him very little, *although his daily work consists in sweeping out a large school-house of seventeen rooms.*

Dr. J. M. Selfridge—That is a very good case. I have frequently verified the truth of Hahnemann's advice in Section 236, viz., "to administer the medicine a short time after the termination of the paroxysm."

Dr. Ledyard had also repeatedly followed the above advice successfully. He had heard of a case in which a large dose of Quinine given *during the paroxysm,* was speedily followed by death.

Dr. Augur—Had a case which called for *Chin-sulph.* (*Quinine*); gave two doses of the 200th potency the following day, and had no return. But, to return to Section 235, latter part, where Hahnemann says that "The symptoms

*during the intermission* should chiefly be taken as guides in selecting the most striking homeopathic remedy." I don't see how that is so.

Dr. Martin—The symptoms occurring during the intermission are very important, because, in intermittents, chill, fever, and sweat are so similar to each other.

Dr. Augur—The chill, fever, and sweat may vary very much. In Note 122 Hahnemann reminds us that there are "countless forms" of such fevers. He (Dr. Augur) thinks the symptoms occurring *during the attack* are more peculiar than those occurring during the intermission.

Dr. Martin (to Dr. Augur)—Did you ever practice in a malarial country?

Dr. Augur—No.

Dr. Martin—In a malarial country the majority of cases are similar.

Dr. J. M. Selfridge—Yes, that is true. I used to cure such cases with two-grain doses of Quinine, while practicing allopathy.

Dr. Ledyard, referring to Section 239, said: "Allen, in his *Therapeutics of Intermittent Fever,* gives us *one hundred and forty-four different medicines, each of which produces its own peculiar kind of intermittent fever,* with twelve pages of symptoms occurring during the "apyrexia" or intermission.

On motion the Club adjourned to meet at the office of Dr. J. M. Selfridge, 1068 Broadway, corner Twelfth Street, Oakland, on Friday, the 18th inst., at 8 P. M.

